# Automation of an atomic force microscope via Arduino

**DOI:** 10.1016/j.ohx.2023.e00447

**Published:** 2023-06-29

**Authors:** Jesus Gerardo Guerrero-Felix, Javier Lopez-Miras, Miguel Angel Rodriguez-Valverde, Carmen Lucia Moraila-Martinez, Miguel Angel Fernandez-Rodriguez

**Affiliations:** a Department of Applied Physics, Faculty of Sciences, University of Granada, Campus de Fuentenueva s/n, 18071 Granada, Spain; b Faculty of Biology, Autonomous University of Sinaloa, 80010 Culiacan, Sinaloa, Mexico; c Department of Electronics and Computer Technology, Faculty of Sciences, University of Granada, 18071 Granada, Spain

**Keywords:** Arduino, Low-cost automation, Atomic force microscope

## Abstract

The Atomic Force Microscopy is a very versatile technique that allows to characterize surfaces by acquiring topographies with sub-nanometer resolution. This technique often overcomes the problems and capabilities of electron microscopy when characterizing few nanometers thin coatings over solid substrates. They are expensive, in the half million dollar range for standard units, and therefore it is often difficult to upgrade to new units with improved characteristics. One of these improvements, motorization and automation of the measurements is very interesting to sample different parts of a substrate in an unattended way. Here we report a low cost upgrade under 60 $ to a Dimension 3000 AFM based on a control unit using an Arduino Leonardo. It enables to acquire dozens or hundreds of images automatically by mimicking keyboard shortcuts and interfacing the AFM PCI card.

## Graphical abstract

**Figure:**
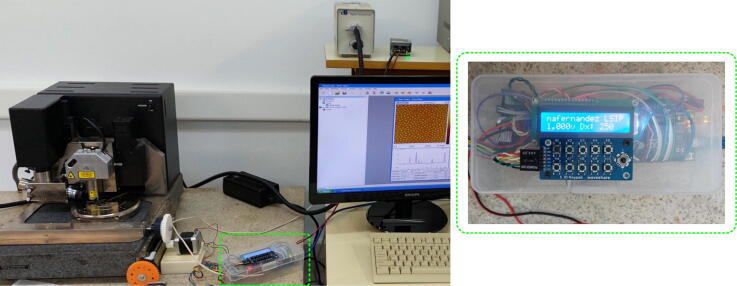

## 1 .

**Specifications table**

**Table:** 

**Hardware name**	Automated AFM
**Subject area**	• *Engineering and material science*
**Hardware type**	• *Imaging tools*
**Closest commercial analog**	*Motorized Dimension Icon Atomic Force Microscope*
**Open source license**	CC BY-SA 4.0

**Cost of hardware**	< 60 $
**Source file repository**	Zenodo [1]

## 2 Hardware in context

**Fig. 1:**
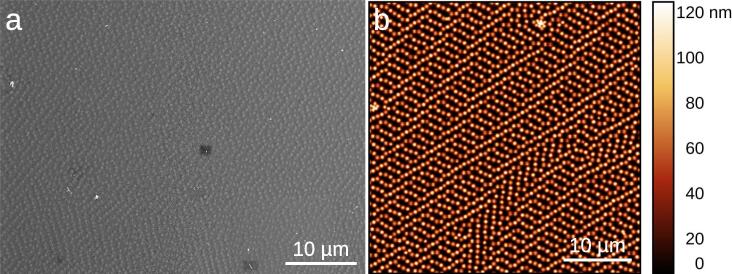
Differences between scanning electron microscopy (SEM) and Atomic Force Microscopy (AFM). Images of a polymeric coating of microgels on a silicon wafer substrate by (a) SEM (GeminiSEM, ZEISS), and (b) AFM (Icon Dimension, Brucker).

Since Binnig, Quate, and Gerber developed the Atomic Force Microscope (AFM) in 1986 [2], it has become a versatile tool in many fields of Science, reflected in being cited more than 10900 times. In particular, in Material Science it enables to characterize materials on solid substrates that are barely or not even visible with electron microscopy due to the very thin size, in the few nanometers. Moreover, the AFM provides a quantitative and precise measurement of the height of the features in the image with precision below one nanometer. The substrate does not need to be metal coated, as it is the case in electron microscopy (see Fig. 1), and it can measure in interesting conditions such as with the sample immersed in liquid, especially interesting for biological samples. The AFM equipment is usually sold in the half million dollars range for standard units, with just a few brands to choose from, and therefore it becomes very expensive to upgrade old but reliable units already working in our labs. We show that it is possible to motorize a 20 years old manual Dimension 3000 AFM via Arduino with sub-micron size positioning accuracy. Such automations are very valuable as experimentalists [3]. Furthermore, with some tweaking it is also possible to automate the measurements such that dozens of AFM images can be taken in an unattended way sampling different positions over the same substrate. It is worth mentioning that although the equipment is 20 years old, it was luckily upgraded to Windows XP several years ago. A quotation for an upgrade to the latest software and a new PC unit was within 10000 $. Such quotation just for the PC unit points out the difficulty for many laboratories to partially upgrade these equipment. Our automation, under 60 $, offers a huge difference in cost to labs compared to acquiring new equipment with similar capabilities, both helping the majority of laboratories that do not have the required funds, and giving a second life to reliable equipment adapted to the new necessities in Science.

## 3 Hardware description

**Fig. 2:**
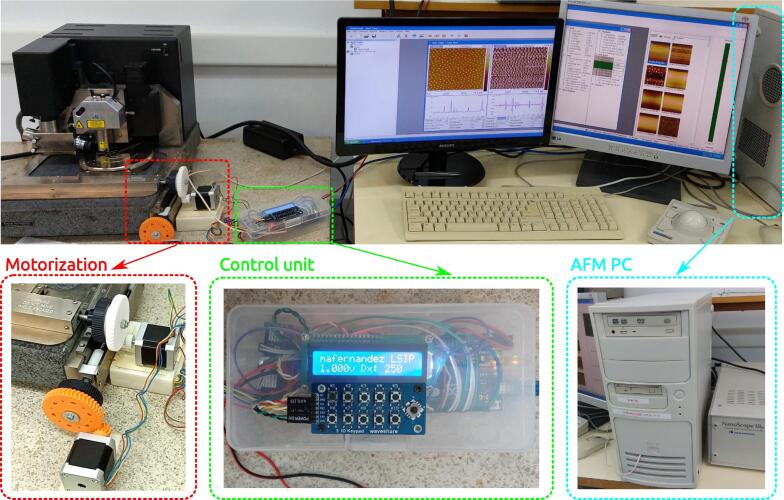
Image of the AFM showing a detail of the motorization, the Arduino based control with automated acquisition, and the PC unit which Arduino interfaces as a keyboard to emulate keyboard shortcuts, and to listen to the beep when the AFM starts the acquisition, the only feedback available from the AFM.

The hardware is described in Fig. 2, and it is comp7811osed of a 20 years old manual Dimension 3000 AFM, a motorization via 3D printed plastic gears and NEMA 17 stepper motors, and an Arduino Leonardo to control this motorization in a manual way via an in-built keyboard with joystick. This Arduino control unit is also used to automate the acquisition and movement by emulating a keyboard when connected via USB to a PC, a feature available in Arduino Leonardo. The gears ratio and the microstepping result in individual steps of 0.18±0.01μm. Since the PC unit is obsolete, we were not able to connect to the serial port of the Arduino to exchange the necessary commands. Instead, it is possible to control all the necessary steps within the AFM commercial software by keyboard shortcuts emulated by the Arduino Leonardo, that is connected via USB to the PC unit. Moreover, the acquisition process involves the probe tip of the AFM approaching physically the sample until it senses that touched it, by measuring the deflection of the probe tip. This process takes different amounts of time for each image taken, and the only feedback coming from the AFM when the acquisition begins is a beep coming from a buzzer attached to a dedicated PCI card. Once the acquisition starts, the time it takes to acquire it is known from the AFM setup and is an input parameter in the Arduino control unit. Thus, with the emulated keyboard from the Arduino Leonardo to control the PC unit, and the feedback through listening to the buzzer to know when the image starts to be acquired, we were able to implement fully unattended automation in which it is possible to take an arbitrary number of images on a sample spaced an arbitrary amount between them. Thus, this low cost motorization and automation extends the features and life of a 20 years old but reliable AFM equipment to scan samples in multiple places in an unattended way.

• It extends the life and capabilities of a 20 years old but reliable Atomic Force Microscope by motorizing it with sub-micron positioning accuracy and by fully unattended automation of the measurements.
• The cost is negligible in comparison to upgrading to a newer Atomic Force Microscope with similar capabilities.
• It can be easily programmed in Arduino to extend the capabilities respect to commercial units thanks to the Open Source firmware and hardware.

### 3.1 Design files

**Fig. 3:**
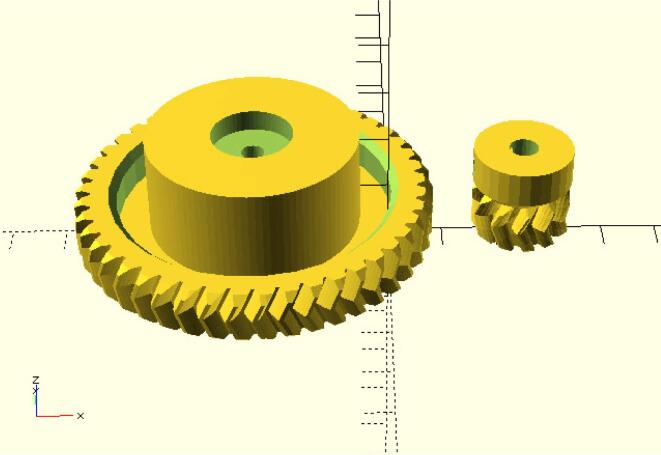
Gears designed with the OpenSCAD Herringbone Wade’s Gears Script.

The gears were designed with the OpenSCAD Herringbone Wade’s Gears Script: [4] (see Fig. 3 and 3D printed with polylactic acid (PLA) plastic filament with a Creality Ender 3 printer. The only parameters tweaked in order to fit the big gears in the AFM were the following: gear2_shaft_diameter = 4.2 mm, gear2_shaft_height = 17.5 mm, gear2_middle_diameter = 39 mm, gear2_middle_rounding  = 0.01 mm, gear2_nut_diameter = 15 mm, gear2_nut_sunk = 4 mm, and gear2_decoration_solid = true. The OpenSCAD source file and the.stl are available in a Zenodo repository [1].

**Fig. 4:**
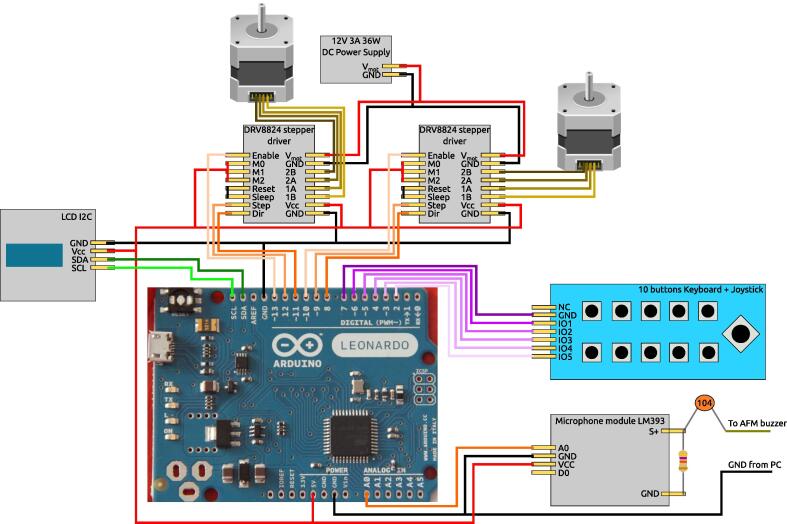
Electronic layout of the automated AFM. Image of an Arduino Leonardo from Wikimedia commons under CC BY-SA 4.0 license.

*Electronics:* *The electronic layout is the one in* Fig. 4 *.*

*Software and firmware:* *Available in a Zenodo repository* *[*1*]* *. The library LCD I2C needs to be installed in Arduino from the library manager, and the 5IOKeypad Arduino Library can be installed manually from the Sourceforge webpage* *[*5*]* *.*

## 4 Design files summary

**Table:** 

**Design filename**	**File type**	**Open source license**	**Location of the file**
Demonstration_AFM	Video	CC-BY-SA	https://doi.org/10.5281/zenodo.7845304
Gears_Automation_AFM_ Arduino	STL file	CC-BY-SA	https://doi.org/10.5281/zenodo.7845304
Gears_Automation_AFM_ Arduino	CAD file	CC-BY-SA	https://doi.org/10.5281/zenodo.7845304
involute_gears	CAD file	CC-BY-SA	https://doi.org/10.5281/zenodo.7845304
teardrop	CAD file	CC-BY-SA	https://doi.org/10.5281/zenodo.7845304
Automation_AFM	INO file	CC-BY-SA	https://doi.org/10.5281/zenodo.7845304

**Demonstration**_**AFM.mp4** – video provides a description of how to perform automated measurement. **Gears**_**Automation**_**AFM**_**Arduino.stl** – file provides 3D model to print the gears. **Gears**_**Automation**_**AFM**_**Arduino.scad** – OpenSCAD file providing the script for generating the 3D gears. **involute**_**gears.scad** - OpenSCAD file providing the script for generating parametric involute bevel and spur gears by GregFrost. **teardrop.scad** - OpenSCAD file providing the script for generating a teardrop shape at the appropriate angle to prevent overhangs greater than 45 degrees. **Automation**_**AFM.ino** – Arduino code for control unit.

## 5 Bill of materials summary

**Table:** 

**Designator**	**Component**	**Number**	**Cost per unit**	**Total cost**	**Source of materials**	**Material type**	
Part #1	Arduino Leonardo	1	21.60 $	21.60 $	Arduino Store	Composite	
Part #2	Set of gears	2	1.05 $	2.1 $	3D printed	Plastic	
Part #3	Stepper motor driver DRV8825	2	5.42 $	10.84 $	Mouser	Composite	
Part #4	Microphone module LM393	1	1.79 $	1.79 $	Solectro	Composite	
Part #5	Ceramic capacitor 100 nF	1	0.188 $	0.188 $	Mouser	Composite	
Part #6	Electrolitic capacitor 100 μF 25 V	1	0.338 $	0.338 $	Mouser	Composite	
Part #7	Resistor 4.7 kΩ	2	0.014 $	0.028 $	Mouser	Composite	
Part #8	Perfboard	1	0.75 $	0.75 $	Solectro	Composite	
Part #9	Female Strip 20 Short Pins	1	0.39 $	0.39 $	Solectro	Composite	
Part #10	Cables male to male 40 cm	1	2.40 $	2.40 $	Bricogeek	Composite	
Part #11	Male 40 Short Pins	1	0.69 $	0.69 $	Solectro	Composite	
Part #12	1602 LCD IIC/I2C	1	2.19 $	2.19 $	Electro componentes	Composite	
Part #13	10 Button Keyboard Module + Joystick	1	3.49 $	3.49 $	Solectro	Composite	
Part #14	Plastic cases	2	1 $	2 $	Hardware store	Plastic	
Part #15	Cable USB 150 cm	1	1.72 $	1.72 $	Super Parts	Composite	
Part #16	Power supply 12 V 3A 36 W	1	5.45 $	5.45 $	EfectoLED	Composite	

*Parts marked with an asterisk were 3D printed on PLA, and estimated at 42* $*/kg.*

## 6 Build instructions

**Fig. 5:**
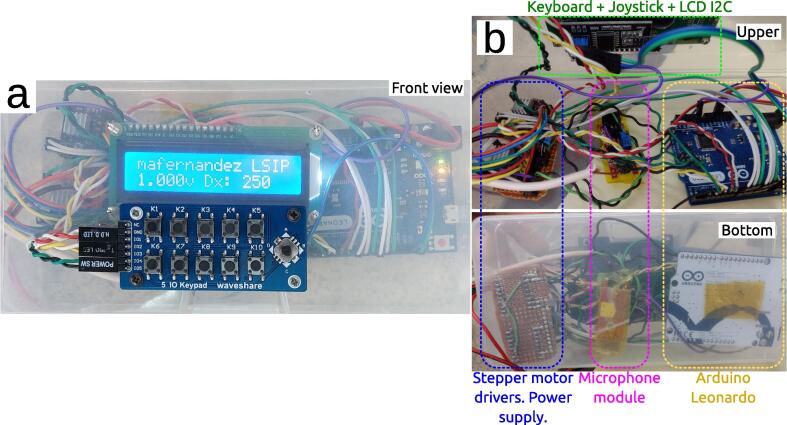
(a) Front view of the control unit where we see the keyboard, joystick and the LCD screen. (b) Inside of the control unit with upper and bottom views of the components.

While this project would benefit from creating a dedicated shield for Arduino, it is still a work in progress, and therefore the electronic schematics in Fig. 4 were mounted on perfboard for the stepper motor drivers and common 5 V, 12 V and ground lines, and everything was encased in a plastic box for protection of the electronics as it can be seen in Fig. 5. As explained in the Hardware Description, the dedicated buzzer from the AFM PCI card cannot be monitored nor controlled from the operative system command console, since it is a different buzzer than the one in-built in the motherboard. Therefore, the workaround was to physically “listen” to this beep by connecting a microphone module directly to the wires of the dedicated buzzer, to look for the millivolts change in the voltage signal. This is performed through the cable that appears from the bottom front of the PC unit in Fig. 2 that goes into a microphone module in the Arduino control unit visible in Fig. 4, Fig. 5, in which the microphone itself was removed and replaced by a 4.7 kΩ resistor. Moreover, the dedicated buzzer had an unusual way of working in which both wires were at 5 V in the idle state. This was solved by decoupling the DC signal through a 100 nF ceramic capacitor to listen only to the AC signal during the beep. Since each AFM will be different, even for different models of the same manufacturer, it is not worth to describe the particular installation of the gears that motorize the manual control, secured in our case by double side adhesive tape. We point the reader to Fig. 2 to get an idea of how to mount and adapt the motorization to their particular AFMs.

## 7 Operation instructions

**Fig. 6:**
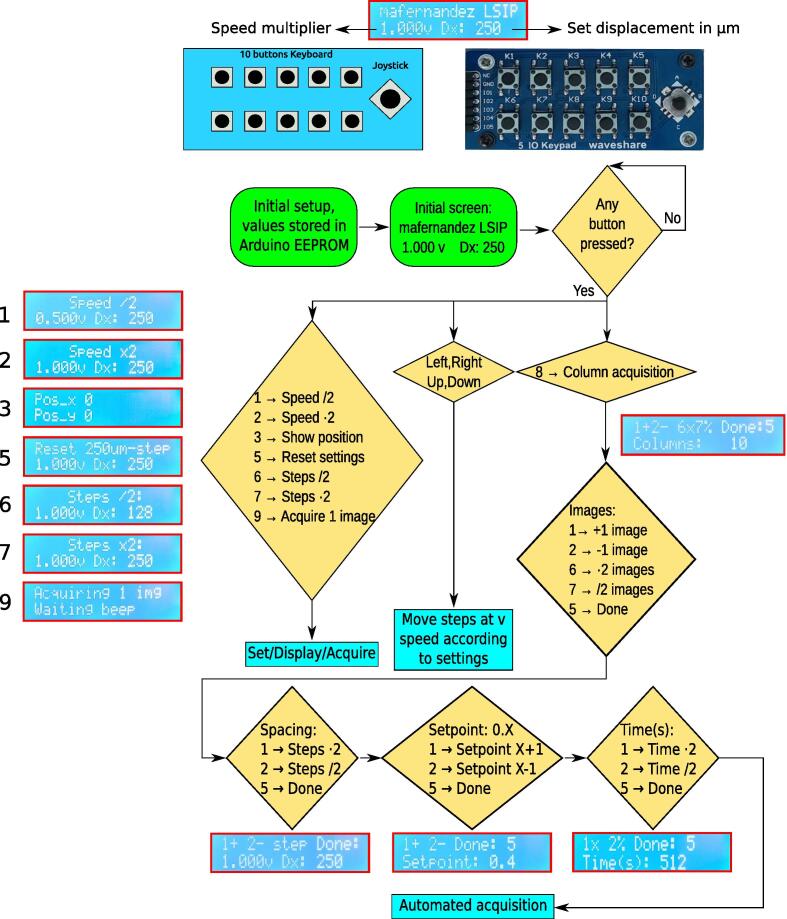
Flowchart of the operations that can be performed with the automated AFM. Inside the red boxes we see the LCD display with the selected operation.

To get started, the user switches on the AFM PC which powers the Arduino Leonardo through the USB port, and the control unit turns on with the initial screen, as shown in Fig. 6 and in the video provided in the additional repository [1]. Next, the user switches on the 12 V power supply that provides the power to the stepper motors. This separation from the rest of the power supply can be useful to implement a panic button for the motorization. In this condition, the motorization is ready and the user can move freely over the sample with the joystick. Depending on the planned work, if the user wants to acquire a few images manually, it is advisable to use the AFM software Nanoscope Version 6 or above, since it is more user friendly. However, in that version not all the required keyboard shortcuts are available, and therefore in order to use the automated acquisition the user needs to open the Nanoscope Version 5 or below. Moreover, the user needs to move the joystick towards the right to account for the backlash present in the AFM threaded rods. This backlash results in limitations discussed in the following sections, and that is worth of trying to mechanically fix it in the future. The movement is to the right because this is the direction in which the automation will move the motors between image acquisitions, and this first movement will make sure the backlash is not a problem regarding the accuracy of the positioning. This is also the reason that for now the automation is only available to acquire images in one column, as there is no possibility to change directions without backlash. Next, the AFM is calibrated as usual, and once the AFM is ready to work with the substrate in place the user will start acquiring an image to make sure all parameters are fine, since these will be used for all further image acquisitions. Once the image is acquired and the tip is withdrawn from the sample, the user must press button 8 on the control unit to start selecting the automation parameters as shown in Fig. 6 and in the video provided in the additional repository [1], such as the number of images, spacing between acquisitions in terms of micrometers, target amplitude setpoint and acquisition time for each image. The later can be calculated from the parameters set in the AFM software, e.g. 512 lines at 1 Hz will take a total of 512 s (this parameter is usually set as powers of 2). Once everything is set within the control unit, the Arduino Leonardo will start to control the AFM, autotuning the tip, approaching the sample until it registers the “beep”, then waiting the set acquisition time and moving to the next position to repeat the process and acquire the next image, repeating it as many times as programmed.

## 8 Validation and characterization

**Fig. 7:**
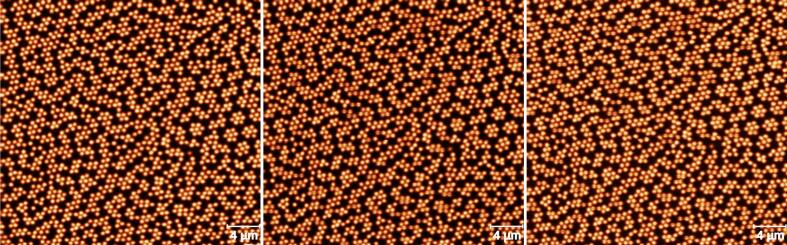
AFM images of a polymeric microgel nanostructure deposited on a silicon substrate acquired sequentially on the same region to show the inherent displacements of the AFM. These displacements are easy to spot by eye at the corners of the images where differences between images are more apparent.

The original AFM positioning system already presents a dispersion in X and Y values even when we measure at the same place, engaging and withdrawing the AFM tip between consecutive image acquisitions. In order to characterize these inherent displacements, we acquired 3 consecutive 32 × 32 μm^2^ images at the same place of a polymeric microgel nanostructure deposited on a silicon substrate, following the protocol described in a previous publication [6] (see Fig. 7). This allowed us to identify single microgels and measure their displacement with sub-micron accuracy between consecutive images. This resulted in displacements ΔX=0.32±0.15μm and ΔY=0.42±0.18μm. Therefore, this sets a lower boundary for the repeatability of our positioning system, in the range of ≃0.5μm.

**Fig. 8:**
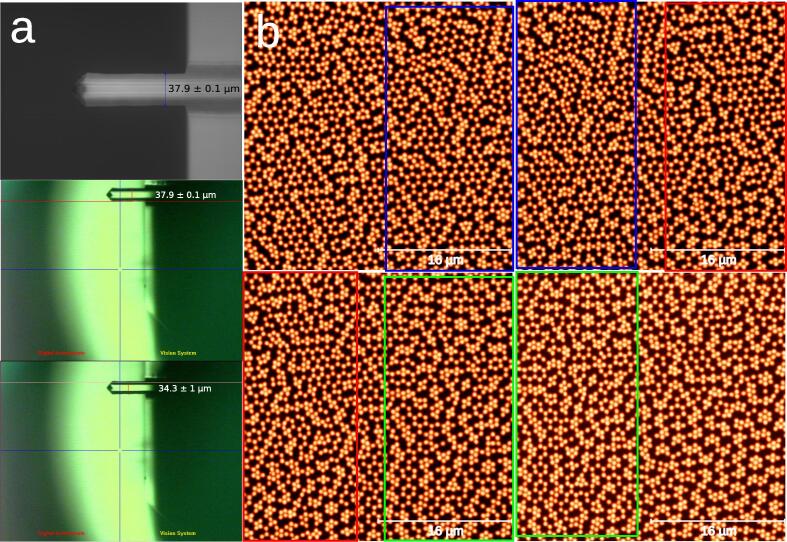
(a) AFM tip imaged by a 50× objective in an optical microscope (top) and imaged by the AFM optical camera (bottom) before and after a 32μm set vertical displacement. (b) AFM 32 × 32 μm^2^ images acquired automatically over the same substrate coated with microgels with 16μm set horizontal displacements between consecutive images. The rectangles of same color correspond to same regions of the substrate.

In order to calibrate the displacement of the individual micro-steps, first a microscope calibration slide with square features was used as a substrate. The motor was moved until the feature disappeared from the optical image. This resulted in an estimated 2.4mm per full round of the big gear. In order to obtain the estimated μm per micro-step we divided the 2.4mm by 200·16 micro-steps, and multiplied for the 11/45 gear reduction factor, since we operate the small gear. This was used as a starting approximation of ≃0.18μm per micro-step. It is worth noting that for this measurement we first displaced the motor in the measuring direction to account for the backlash present in the AFM threaded screws. The backlash, over 100μm when switching directions of the motor, is a problem in terms of accuracy in positioning if the joystick is moved in different directions each time, but as long as the motors move in the same direction after a few first movements that correct the backlash, the rest of movements are accurate as discussed. Next, we used an AFM tip that we characterized in an optical microscope with a 50x objective (WLCM, PLμ Confocal Imaging Profiler, Sensofar) resulting in a width of 37.9±0.1μm (see Fig. 8a). Then, we placed the tip in our AFM and focused it and displaced the stage 32μm and observed how much it displaced over the optical camera images (see Fig. 8a), this resulted in 34±1μm. Given the low quality of the AFM optical camera, we decided to acquire AFM images of the same sample in Fig. 7. Therefore, by following the Operation instructions described before, we automatically acquired eight 32 × 32 μm^2^ images with a set X-displacement between images of 16 μm, an amplitude setpoint of 0.9 V, and 512 s acquisition time for each image (see Fig. 8b showing 4 of the 8 acquired images). Thanks to this automation, an arbitrary number of images can be acquired in an unattended way. As described above, we identified single microgels in the AFM images and quantified how much they moved compared to the pre-set 16μm displacement in X (squared regions of same color between consecutive AFM images in Fig. 8b correspond to same regions of the substrate), resulting in ΔX=17.8±0.4μm and ΔY=0.22±0.16μm. Thus, the displacement in Y was within the inherent positioning error of the AFM as discussed above, and the displacement in X was only slightly above the initial guess, 17.8±0.4μm instead of 16μm.

With this information, we corrected our initial calibration as 0.18μm·17.8μm/16μm=0.20μm per micro-step, and expected a minimum error in positioning between images in the range of ≃0.5μm, inherent to the AFM original positioning system. By acquiring a new set of 8 images (not shown) we found that the displacement between images was reduced to ΔX=0.7±0.4μm, compatible with the inherent displacements of the AFM described above. It is worth noting that this is a remarkable positioning accuracy for such an inexpensive setup.

Possible mechanical improvements would include mechanically upgrading the original AFM positioning system to reduce the significant backlash present in the system, over 100μm when switching directions of the motor. Currently, we restrict this source of inaccuracy by moving the motors in one way during automated acquisition as described before. Reducing the backlash by other mechanical means would allow automated acquisitions in two dimensions without restrictions. This would be a very important advantage when carrying out consecutive measurements requiring large area characterizations. An easier upgrade would be including Wi-Fi to the Arduino to operate the unit remotely. In this sense, the Arduino platform allows for many possible upgrades with little extra effort.

## CRediT authorship contribution statement

**Jesus Gerardo Guerrero-Felix:** Investigation, Data curation, Visualization, Writing – original draft, Writing – review & editing. **Javier Lopez-Miras:** Investigation. **Miguel Angel Rodriguez-Valverde:** Funding acquisition, Writing – review & editing. **Carmen Lucia Moraila-Martinez:** Funding acquisition, Writing – review & editing. **Miguel Angel Fernandez-Rodriguez:** Conceptualization, Methodology, Software, Validation, Writing – original draft, Writing – review & editing, Supervision, Funding acquisition.

## Declaration of Competing Interest

The authors declare that they have no known competing financial interests or personal relationships that could have appeared to influence the work reported in this paper.
